# Preventive and therapeutic opportunities: targeting BAP1 and/or HMGB1 pathways to diminish the burden of mesothelioma

**DOI:** 10.1186/s12967-023-04614-5

**Published:** 2023-10-25

**Authors:** Michele Carbone, Michael Minaai, Yasutaka Takinishi, Ian Pagano, Haining Yang

**Affiliations:** Thoracic Oncology, University of Hawaii Cancer Center, 701 Ilalo St, Honolulu, HI 96813 USA

## Abstract

Mesothelioma is a cancer typically caused by asbestos. Mechanistically, asbestos carcinogenesis has been linked to the asbestos-induced release of HMGB1 from the nucleus to the cytoplasm, where HMGB1 promotes autophagy and cell survival, and to the extracellular space where HMGB1 promotes chronic inflammation and mesothelioma growth. Targeting HMGB1 inhibited asbestos carcinogenesis and the growth of mesothelioma. It is hoped that targeting HMGB1 will be a novel therapeutic strategy that benefits mesothelioma patients. Severe restrictions and/or a complete ban on the use of asbestos were introduced in the 80 and early 90s in the Western world. These measures have proven effective as the incidence of mesothelioma/per 100,000 persons is decreasing in these countries. However, the overall number of mesotheliomas in the Western world has not significantly decreased. There are several reasons for that which are discussed here: (1) the presence of asbestos in old constructions; (2) the development of rural areas containing asbestos or other carcinogenic mineral fibers in the terrain; (3) the discovery of an increasing fraction of mesotheliomas caused by germline genetic mutations of BAP1 and other tumor suppressor genes; (4) mesotheliomas caused by radiation therapy; (5) the overall increase in the population and of the fraction of older people who are much more susceptible to develop all types of cancers, including mesothelioma. In summary, the epidemiology of mesothelioma is changing, the ban on asbestos worked, there are opportunities to help mesothelioma patients especially those who develop in a background of germline mutations and there is the opportunity to prevent a mesothelioma epidemic in the developing world, where the use of asbestos is increasing exponentially. We hope that restrictive measures similar to those introduced in the Western world will soon be introduced in developing countries to prevent a mesothelioma epidemic.

## Epidemiology

Mesothelioma is a cancer that has been tightly linked to the use of asbestos, a commercial name used to identify 6 among over 400 mineral fibers present in the natural environment, that were mined for commercial use [1]. About 4.6% of asbestos miners who worked in mines for at least 10 consecutive years [2] and close to 7–8% of insulators and pipefitters who used asbestos without precautions in the 60 and 70s developed mesothelioma [3]. In these workers, mesothelioma developed with a latency of 20–60 years from exposure, evidence of a chronic carcinogenic process [4].

Because of the link between asbestos exposure and mesothelioma, in the 80s, the use of asbestos was severely restricted (USA) or entirely banned (Western Europe, Australia, and a few more countries) [5, 6]. As expected, 40 years or so later, the asbestos ban led to a significant declining trend in the incidence of diffuse malignant mesothelioma in the industrialized countries that implemented a ban or restrictive measures on the use of asbestos [7–9].

The decrease in incidence of mesothelioma has been seen only in males, which is expected since males are much more frequently occupationally exposed to asbestos than females [9]. Accordingly, while in the past in industrialized countries mesothelioma was diagnosed mostly in workers involved in the asbestos trade, presently mesothelioma is often diagnosed in patients who have not been occupationally exposed to asbestos [10]. Some of these patients may have been exposed to asbestos present in old buildings or constructions. Others may have been exposed to asbestos or to other carcinogenic fibers, such as erionite and antigorite that are naturally present in the environment. As rural areas containing these fibers are being developed, construction workers first and residents later can be exposed. In some cases, these exposures can be above background levels and may cause lung fibrosis, pleural plaques, and mesothelioma [3, 6, 9, 11–14].

In addition, mesothelioma can develop in carriers of germline mutations of BAP1 and of other tumor suppressor genes. BAP1 mutations cause prevalently mesothelioma and melanomas. Mutations of other tumor suppressor genes, for example, TP53, BRCA1, BRCA2, etc., cause prevalently other cancer types but may also cause mesothelioma [9, 15–19]. Some germline mutations, such as BAP1, are very powerful and may cause mesothelioma and other malignancies in the absence of environmental exposure to asbestos or to other carcinogens [20], others, may cause mesothelioma prevalently when associated with environmental exposure to asbestos or other carcinogenic fibers [3, 18, 21]. Presently it has been estimated that approximately 12% of all mesotheliomas are linked to germline mutations of BAP1 or other tumor suppressor genes; presumably in the past, these mesotheliomas were attributed exclusively to asbestos [9, 15–19].

In addition, a new category of mesothelioma patients has emerged in recent years. These patients were treated and cured with radiation therapy for lymphomas, seminomas, ovarian carcinomas, etc. Several years later, these patients may develop mesotheliomas, angiosarcomas, and other malignancies in the areas that were irradiated [22, 23].

In summary, in the industrialized world, the epidemiology of mesothelioma has been changing in recent years. The measures taken in the 80 and 90s to restrict or ban asbestos use did, as anticipated, save lives by decreasing the incidence of mesothelioma [7–9]. However, while the incidence of mesothelioma × 100,000 persons is decreasing, the overall number of mesotheliomas has not declined significantly, at least not in the USA as the total population is increasing. Presently mesotheliomas are occurring for a combination of several factors: (1) the presence of asbestos in old constructions that in some cases can result in substantial exposure; (2) the development of rural areas containing asbestos and other carcinogenic fibers, which have caused exposure in workers first and residents later [9, 12–14] (3) the discovery that 12–16% of mesotheliomas are linked to germline mutations [15–17, 24, 25]; (4) the development and use of radiation therapy in the past decades to treat several malignancies, which may cause mesothelioma years later [22, 23, 26, 27]; (5) in addition, mesothelioma, like most other cancers, affects prevalently old people. The population in industrialized countries is increasing and growing older, and thus the number of people most susceptible to developing mesothelioma is increasing [6]. At times, some of these factors may also interact with each other and promote the development of mesothelioma [9, 21].

At the epidemiological level, mesotheliomas associated with asbestos exposure develop in older individuals with a 10–5:1 M:F ratio and a 10–5:1 pleural: peritoneal ratio (the highest the ratio the highest the percentage of asbestos workers). Mesotheliomas in individuals younger than 55 years, with a M:F ratio close to 1:1, and equally distributed between pleura and peritoneum, are instead often associated with environmental exposure to carcinogenic fibers present in the natural environment—which cause exposure since birth, or they develop in individuals carrying germline mutations of BAP1 or of other tumor suppressors genes [6, 9].

So far, we have underscored “the industrialized world”—in particular, the USA, Australia, and Western Europe, where the use of asbestos has been almost entirely banned for about 40 years. How about the developing countries, where, instead, the use of asbestos continues to increase exponentially [3]?

Some studies have tried to study the incidence of mesothelioma in developing countries, but the results are difficult to interpret. Apparently, despite the extensive use of asbestos, the incidence of mesothelioma in developing countries is very low [3, 7]. However, several factors bias the data. To start, life expectancy is shorter in developing countries: given the long latency between asbestos exposure and the development of mesothelioma, many workers may not reach old age when mesothelioma develops [3]. Moreover, the diagnosis of mesothelioma in developing countries is imprecise: ~ 50% of patients diagnosed as having mesothelioma may not have mesothelioma [28]. It appears likely that the opposite may be even more frequent—i.e., a patient with mesothelioma diagnosed with another cancer or medical condition. This is due to a lack of awareness and training to diagnose mesothelioma and a lack of the immunohistochemically and/or electron microscopic tools that are required to diagnose this malignancy with confidence [3, 29].

Since in developing countries, the use of asbestos is increasing, it seems reasonable to expect that the incidence of mesothelioma will increase. Therefore, as we noted in a recent commentary [9], we suggest that restrictive measures similar to those introduced in the Western world should be introduced in developing countries before these nations are faced with a mesothelioma epidemic.

## Mechanisms of carcinogenesis of asbestos and of other mineral fibers

These mechanisms remained elusive for many years. When added to human mesothelial cells in tissue culture, asbestos is cytotoxic and the entire cell population is wiped out in a matter of a few days. This is because mesothelial cells attempt to phagocytize asbestos fibers and die in the process [30]. In these tissue culture experiments, it is often possible to see asbestos fibers “perforating’ mesothelial cells, sometimes perforating the nuclei of these cells. Some of these cells may attempt to divide while showing asbestos fibers going through their nuclei. This led to the hypothesis that asbestos could cause cancer by mechanically causing genetic damage [3]. However, we have never seen primary human mesothelial cells exposed to asbestos alone survive [3, 30, 31]. Indeed, transformed cell lines were never derived from such experiments.

Recent studies indicate that asbestos/fiber carcinogenesis occurs because of the chronic inflammatory process that follows the deposition of mineral fibers in tissues. Indeed, the most biopersistent fibers, such as crocidolite asbestos and erionite, are also the most carcinogenic [3, 4, 31]. It was found that mesothelial cells exposed to asbestos or other carcinogenic fibers die and release HMGB1 estracellularly [32]. HMGB1 is a nuclear protein that normally protects DNA from damage, when released extracellularly, it kicks start an inflammatory process characterized by the release of TNF-α and of other cytokines, which in turn attract granulocytes, monocytes, and tissue macrophages. These cells actively secrete HMGB1 extracellularly together with various cytokines and mutagenic reactive oxygen species that can cause DNA damage. This inflammatory process becomes chronic because carcinogenic fibers cannot be easily removed from tissues. In addition, as HMGB1 moves from the nucleus into the cytoplasm, it activates autophagy, processes that help mesothelial cells survive asbestos exposure [1, 32–35]. Eventually, over the course of many years in humans, and in a period of 9–18 months in mice, this chronic inflammation and accumulation of genetic damages may lead to the development of mesothelioma [1, 3, 31–35].

The link between chronic inflammation HMGB1 and mesothelioma, is underscored by the finding that mineral fibers that do not elicit HMGB1 release and thus a sustained chronic inflammatory process, such as palygorskite a mineral fiber abundantly present in in Nevada, are not carcinogenic [36].

Because of the field effect of asbestos and of other carcinogenic fibers, several foci of cell transformation may occur over the serosal membranes, accordingly, mesotheliomas are often polyclonal [37].

Possibly because mesotheliomas emerge from an environment rich in HMGB1, mesothelioma cells often actively secrete HMGB1, a process that promotes mesothelioma progression and invasion [38]. Drugs that inhibit HMGB1 directly (such as BoxA or monoclonal antibodies) or indirectly (such as aspirin [39] or ethyl pyruvate [40]) inhibit the growth of mesothelioma in mice and the growth of human mesothelioma cell lines in tissue culture [35, 38, 41, 42]. This has led to the hypothesis that developing a specific HMGB1 inhibitor for human use should help patients with mesothelioma [35, 38, 41, 42]. Alternatively, targeting NF-κB may reduce HMGB1 activity and impair mesothelioma growth [43, 44].

Asbestos synergizes with SV40, a DNA tumor virus that causes mesothelioma in hamsters, in causing the malignant transformation of human mesothelial cells in tissue culture, and in causing mesothelioma in rodents [30, 45, 46]. However, because of the absence of supporting epidemiological data, the possible relevance of this interaction to the development of human mesothelioma remains to be demonstrated [47].

## BAP1 and mesothelioma

Studying multiple cases of mesothelioma in Cappadocian families, we discovered that the susceptibility to mesothelioma was transmitted in a Mendelian fashion [48]. Given the 100% penetrance of this condition, we postulated that a genetic factor was responsible for familial mesothelioma. Multiple US families who also had multiple cases of mesotheliomas in their families approached us. The possibility of multiple cases of mesothelioma in a single nuclear family, in the absence of an obvious history of occupational asbestos exposure, is almost zero. Specifically, the overall incidence of mesothelioma in the US is about 3200 cases per year, about 2600 males and about 600 females. The overall probability of developing mesothelioma in the United States is 9.65E−06 (3200/331,449,281). In the US the male/female ratio is 13:3 (2600:600), so the male probability is 1.57E−05 and the female probability is 3.62E−06. Therefore, the probability of having more than one family member developing mesothelioma by chance is extremely low. For example, in a family of 5, 3 males and 2 females, the probability of two males developing mesothelioma is 7.38E−10. With the support of an NCI P01 (M Carbone PI) to identify this putative mesothelioma gene, we focused our studies on two US families with multiple affected males and females in which none of the family members had been occupationally exposed to asbestos. After 4 and half years of manually sequencing “miles and miles” of DNA—this was pre NGS era—we discovered that all individuals affected by mesotheliomas in these families carried truncating mutations of the BAP1 gene [49] and reviewed in [50–52]. Our findings were confirmed by multiple studies [52, 53]. Moreover, we and others found that mutations of additional tumor suppressor genes, including BRCA1, BRCA2, TP53, BLM, etc., predisposed to mesothelioma. Overall, it is estimated that 12–16% of mesotheliomas develop in carriers of germline BAP1 mutations-the most common mutation, or of other tumor suppressor gene mutations [16–19, 25]. Some of these mutations, like BAP1, have 100% cancer penetrance and thus are sufficient to cause cancer, others may increase susceptibility to asbestos and to other carcinogens present in the environment [21, 52].

The reader may find some discrepancies in the literature, as some articles suggested that the incidence of cancer in carriers of germline BAP1 mutations is around 80% [53], while we reported that is close to 100% [52]. The likely reason for this apparent discrepancy is that other studies measured cancer incidence in each family at one point in time. We instead, have been following a total of 98 BAP1 mutant families (as of March 2023) across the US and abroad for many years, some of these families for over 20 years. At any point in time, there will be someone in these families who carry the mutation and is free of tumor, however as we followed these families, we noted that eventually affected family members developed cancer, about 30% of them mesothelioma [15, 52].

## Why are BAP1 inactivating mutations so powerful in causing mesothelioma and other cancers?

**Fig. 1:**
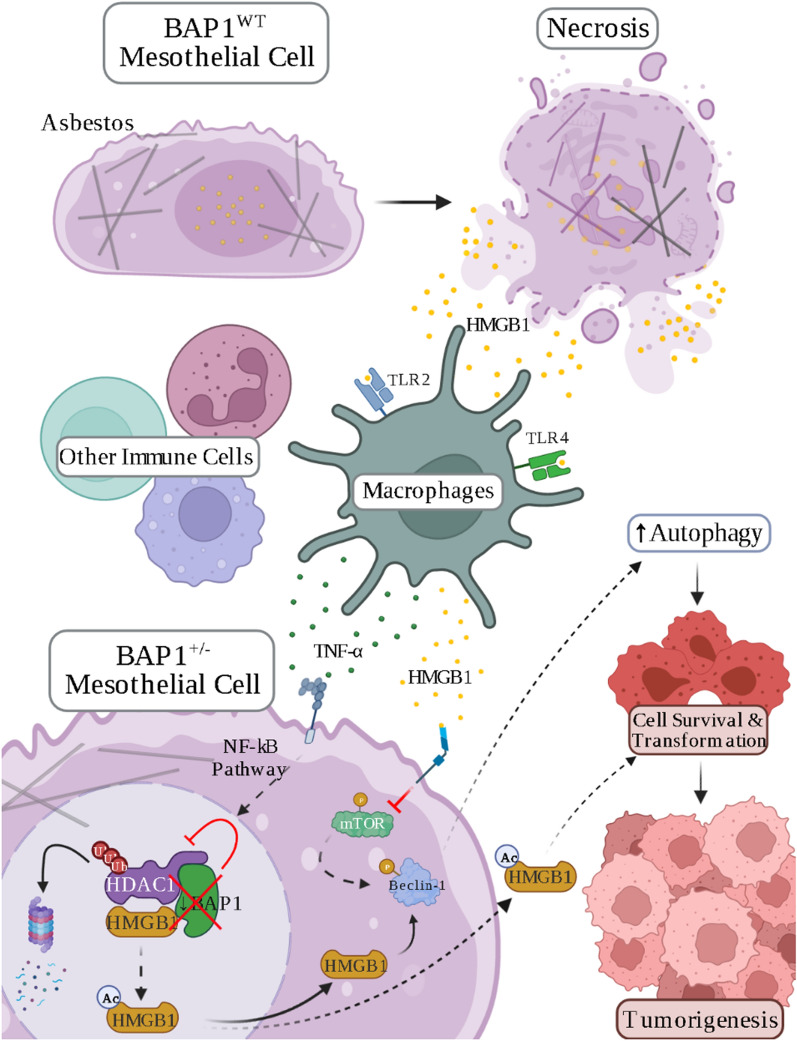
Diagram showing the regulation of chronic inflammation and cell transformation by the BAP1, HDAC1, and HMGB1 trimer. Asbestos causes mesothelial cell death that in turn leads to the release of HMGB1 from the nucleus to the extracellular space, where HMGB1 acts as a DAMP recruiting macrophages and other inflammatory cells. Macrophages actively secrete HMGB1 and TNF-α propagating inflammation around asbestos deposits in tissue, a process that over time can cause malignant transformation and mesothelioma. A similar process takes place in mesothelial cells carrying germline BAP1 mutations (BAP1^+/−^), depicted at the bottom of the figure, even in the absence of asbestos exposure. In BAP1 wild-type (WT) cells, nuclear BAP1 deubiquitylates and stabilizes HDAC1, which deacetylates HMGB1: deacetylated HMGB1 remains in the nucleus. In BAP1^+/−^ cells, the reduced BAP1 levels cause HDAC1 ubiquitylation and degradation. This, in turn, causes increased acetylation of HMGB1. Acetylated HMGB1 moves to the cytoplasm where it activates autophagy, and from there to the extracellular space where HMGB1 propagates chronic information that favors malignant transformation and mesothelioma growth. The combination of asbestos exposure and BAP1 germline mutations can cooperate in propagating chronic inflammation and malignant transformation [21]. Created with BioRender.com

BAP1 is a deubiquitylase, thus its inactivation alters multiple cellular processes [52, 54]. In the nucleus, BAP1 participates in chromatin remodeling and DNA repair [52, 54], in the cytoplasm BAP1 regulates calcium flux [55] and cell metabolism [56]. BAP1 mutations cause a switch from oxidative phosphorylation to aerobic glycolysis [52]. Moreover, BAP1 mutations impair apoptosis [55] and ferroptosis [57]. It has been recently suggested that BRCA1 haploinsufficiency impairs iron metabolism to promote chrysotile-induced mesothelioma via ferroptosis resistance [58]. Two additional mechanisms may account for the association of BAP1 mutations with mesothelioma and its improved survival. We recently discovered that in the nucleus BAP1 binds and deubiquitylates HDAC1, which in turn binds and deacetylates HMGB1, keeping HMGB1 in the nucleus. When BAP1 is reduced by heterozygous mutations or absent because of biallelic inactivation, HDAC1 becomes ubiquitylated and is degraded. This leads to acetylation of HMGB1, which in turn causes the transfer of HMGB1 to the cytoplasm and to the extracellular space where HMGB1 promotes mesothelioma. Therefore, BAP1 mutations activate the same mechanism—HMGB1 nuclear to cytoplasmic to extracellular release—that promotes asbestos-induced mesothelioma [34] (Fig. 1).

## The puzzling marked improved survival of mesothelioma patients carrying germline BAP1 mutations

There is also a positive side to carrying BAP1 mutations: mesotheliomas in these patients are for the most part less aggressive, as they are associated with a median survival of 6–7 years [15–17, 19]. Some patients survived 20+ years, and are either alive or died of old age or other causes. Because BAP1 carriers often develop multiple malignancies, it is critical to enroll these patients, and their affected family members, in screening programs for early detection that can be lifesaving [15].

**Fig. 2:**
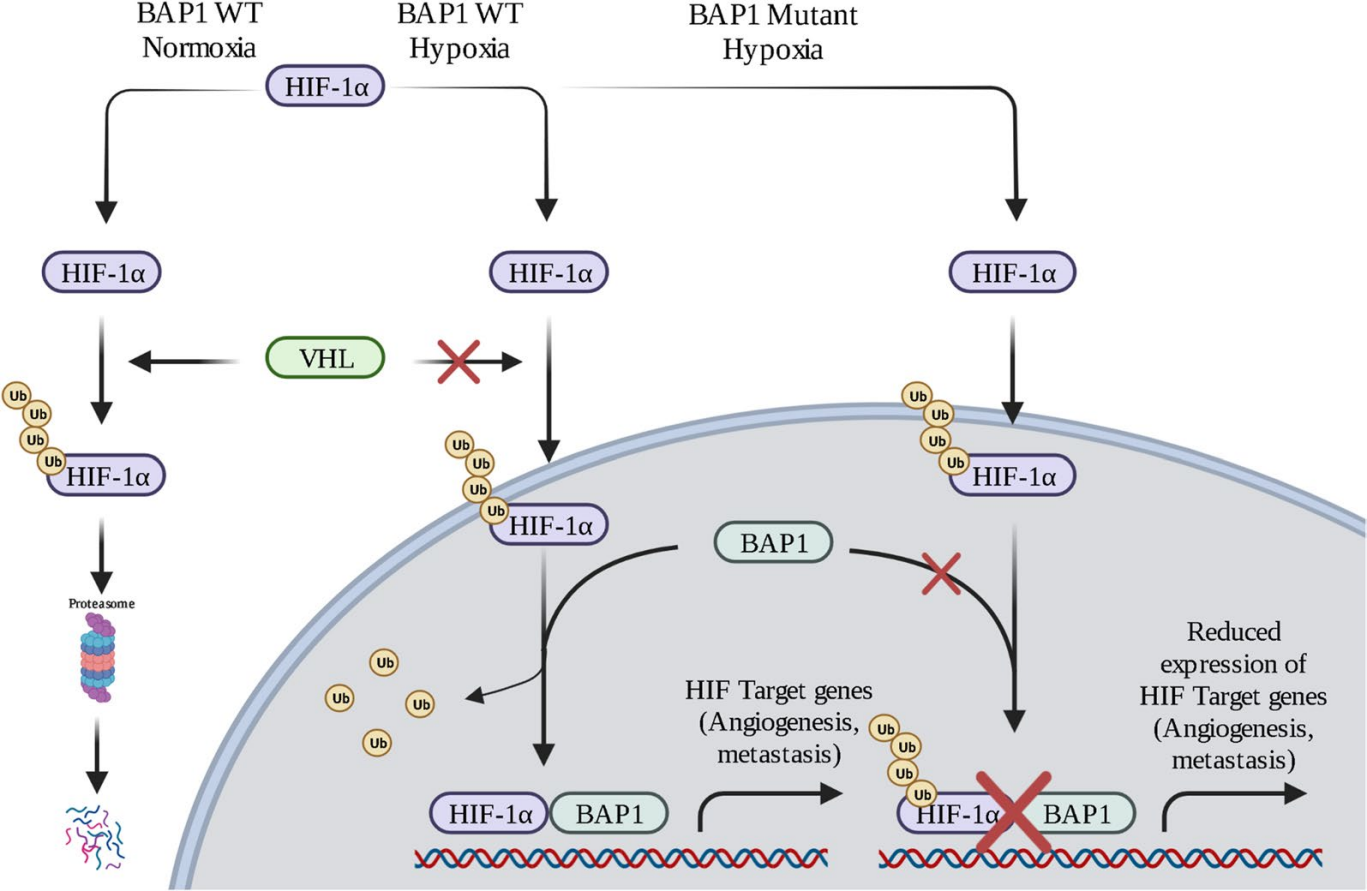
BAP1 regulation of HIF-1α. HIF-1α is a key regulator of the adaptive response to a hypoxic environment and tumor cell invasion. HIF-1α is ubiquitylated in normoxia and it is degraded via an oxygen-dependent mechanism mediated by VHL. In the nucleus BAP1 binds, deubiquitylates, and stabilizes HIF-1α. In BAP1^+/−^ carriers during hypoxia, the lack or reduced presence of BAP1 is accompanied by a significant reduction of HIF-1α which may contribute to the reduced aggressiveness of tumors. Created with BioRender.com

As for the biological reasons that cause the improved survival of mesothelioma patients carrying BAP1 mutations, two very recent discoveries shed some light on this puzzling issue: (1) Louw et al., reported that biallelic inactivating BAP1 mutations increase susceptibility to chemotherapy [59]; and (2) we discovered that BAP1 deubiquitylates and stabilizes HIF-1α. HIF-1α promotes tumor growth and invasion. In the absence of HIF-1α, tumor cells have difficulty in invading nearby tissues and growing in hypoxia (Fig. 2) [60]. This finding provides a novel approach to target HIF-1α to impair tumor growth. We are now exploring the potential therapeutic application of our discovery.

## Acquired (somatic) BAP1 inactivating mutations in mesothelioma

The critical role of BAP1 in the pathogenesis of mesothelioma is underscored by the finding that over 60% of sporadic—not genetically related—mesotheliomas carry biallelic inactivating BAP1 mutations. These mutations can be easily detected by immunohistochemistry as nearly all pathogenic BAP1 mutations are truncating mutations: since the nuclear localization signal is present in the carboxy-terminus of the BAP1 protein, all truncating mutations cause a lack of nuclear staining, while the BAP1 cytoplasmic stain is, at times, retained [15, 52, 61].

*BAP1* and *CDKN2A* are the most common inactivating mutations in mesothelioma. Approximately 60–70% of mesotheliomas contain inactivating *BAP1* mutations, which can be detected using an integrated genomic approach or simply by absence of BAP1 nuclear staining, since almost 100% of BAP1 inactivating mutations cause the loss of the nuclear localization signal located at the carboxy-terminus of the BAP1 protein [61]. Instead NGS or Sanger sequencing detect only ~ 50% of the BAP1 mutations, as these techniques were designed to identify single nucleotide changes and for the most part miss deletions in the range of 300–3000 kb or whole allele deletions which account for about half of BAP1 inactivating mutation [62]. This has resulted in some confusion in the literature as manuscript that relied only on NGS and Sanger sequencing, underestimated the true frequency of inactivating BAP1 mutations. CDKN2A inactivation is present in approximately 50% of mesotheliomas and can be reliably detected by fluorescent in situ hybridization (FISH), or by immunostain for MTAP, a gene that is most frequently co-deleted with the nearby CDKN2A gene [29, 61–63]. BAP1 and CDKN2A are not mutated in benign reactive mesothelial hyperplasia. Accordingly, detection of either absence of nuclear BAP1 immunostain and/or CDKN2A inactivation in lesional cells is now routinely used in diagnostic pathology to distinguish benign reactive mesothelial hyperplasia, including chronic pleuritis, from malignant mesothelioma [29, 64].

## Conclusions

Diffuse Malignant Mesothelioma is a cancer strongly linked to asbestos fibers exposure. The ban and/or very restrictive measures implemented in the Western world on asbestos use have been effective in reducing the incidence of mesothelioma.

The development of remote areas that naturally contain asbestos and other carcinogenic fibers in the soil and asbestos in place in old construction is creating new categories of asbestos-exposed individuals. These sources of exposure should be identified promptly to implement preventive measures to decrease the risk of mesothelioma. Such measures have been implemented, for example, in Cappadocia, Turkey where the Turkish government built two new villages with asbestos and erionite fiber-free material, and relocated the villagers. Similarly, in ND Dakota, the State repaved over 300 miles of clean gravel roads that had been initially paved with erionite-contaminated gravel causing significant exposure to those who built and those who drove over those roads. These measures likely saved many lives from developing mesothelioma.

Radiation therapy to treat malignancies in the chest and abdomen has saved lives but has had the side effect of causing mesotheliomas and various types of sarcomas several years later in these same patients. This “new” group of mesothelioma patients should decrease in the coming decades as improvements in radiation therapy have significantly reduced the field of exposure to precisely match affected areas sparing nearby tissues.

In the recent past, there has been a better appreciation of the role of inherited germline mutations of various tumor suppressor genes in human cancer (BAP1, BRCA1 and 2, TP53, RB, PTEN, ATM, etc.). Among them, BAP1 plays a particular role in mesothelioma. Intriguingly these mesotheliomas have often a much less aggressive course, appear to respond better to therapy, and some patients were cured and died of old age or other diseases. This is the first time in the history of mesothelioma that we see patients cured. We hope that by studying the mechanisms responsible for the less aggressive phenotype of these mesotheliomas, we may be able to target these mechanisms and help all mesothelioma patients. In this regard, it may be helpful to design novel therapies that benefit all mesothelioma patients that asbestos-induced mesotheliomas and mesotheliomas developing in carriers of germline BAP1 mutations share at least some of the same pathogenic mechanisms. Both are promoted by the extracellular release of HMGB1, which may be caused either by exposing cells to asbestos or by BAP1 deficiency. Finally, mesothelioma is cancer, and, like all cancers can develop spontaneously. The older the population, the higher the chance of developing spontaneous malignancies, as cells inevitably accumulate genetic damage as we age. This problem is difficult to solve.

## Data Availability

Data sharing not applicable to this article as no datasets were generated or analyzed during the current study.
